# Targeted Delivery of Bortezomib Using Retinoid-Based Nanoparticle: Modulating Liver Fibrosis through the TGF-β1/Smad3 Pathway

**DOI:** 10.34172/apb.43295

**Published:** 2025-02-12

**Authors:** Samaneh Siapoush, Mohammad Rahmati, Morteza Milani, Behzad Hatami, Nosratollah Zarghami, Abbas Ebrahimi-Kalan, Mohammad Reza Zali, Kaveh Baghaei

**Affiliations:** ^1^Department of Medical Biotechnology, Faculty of Advanced Medical Sciences, Tabriz University of Medical Sciences, Tabriz, Iran.; ^2^Basic and Molecular Epidemiology of Gastrointestinal Disorders Research Center, Research Institute for Gastroenterology and Liver Diseases, Shahid Beheshti University of Medical Sciences, Tehran, Iran.; ^3^Department of Clinical Biochemistry and Laboratory Medicine, Faculty of Medicine, Tabriz University of Medical Sciences, Tabriz, Iran.; ^4^Infectious and Tropical Diseases Research Center, Tabriz University of Medical Sciences, Tabriz, Iran.; ^5^Gastrointestinal Disorders Research Center, Research Institute for Gastroenterology and Liver Diseases, Shahid Beheshti University of Medical Sciences, Tehran, Iran.; ^6^Department of Medical biochemistry, Faculty of Medicine, Istanbul Aydin University, Istanbul, Turkey.; ^7^Department of Neurosciences and Cognition, Faculty of Advanced Medical Sciences, Tabriz University of Medical Sciences, Tabriz, Iran.

**Keywords:** Bortezomib, Proteasome inhibitor, Liver fibrosis, TGF-β1, Nanoparticles

## Abstract

**Purpose:**

Hepatic stellate cells (HSCs) play a crucial role in fibrosis progression. we have developed a targeted delivery approach using A-functionalized nanoparticles for bortezomib (BTZ) specifically for activated HSCs in a mouse model of liver fibrosis.

**Methods:**

The emulsion solvent evaporation method was used to form nanoparticles (NPs) targeted with vitamin A. The characterization of NPs was approved with Fourier-transform infrared (FT-IR), dynamic light scattering (DLS), and scanning electron microscopy (SEM). Also, the biodistribution of NPs inside mice bodies was conducted via fluorescent drug. the cytotoxicity of NPs evaluated in different dose in vitro test. Compared to control groups, a serological evaluation, molecular examination and protein expression were performed based on BTZ‘s impact on fibrotic index on model mice after treatment with targeted NPs loaded with BTZ.

**Results:**

Characterization of synthesized targeted NPs containing BTZ through DLS, X-ray diffraction (XRD) and FT-IR showed that the size of NPs was optimum and drug was entrapped inside of NPs successfully. Biodistribution of engineered targeted nanoparticles incorporating BTZ in mice showed a gradual tendency of NPs in the liver zone. Moreover, mice treated with vitamin A-targeted containing BTZ showed decreased expression of collagen I, collagen III, and α-SMA; also, the level of expression in TGF-β1/Smad3 and nuclear factor-kappa B (NF-κB) genes suppressed in mice treated with NPs entrapped BTZ. In line with these results, histopathologic and serological results showed significant exacerbation in non-target and drug-free nanoparticle-treated mice. The best result was seen in mice treated with targeted BTZ.

**Conclusion:**

BTZ, in low amounts entrapped in targeted NPc, could ameliorate the fibrotic index in mice models.

## Introduction

 Hepatic fibrosis is defined as the replacement of functional liver tissue with excessive extracellular matrix (ECM) protein accumulation, leading to vascular architectural distortion and liver dysfunction.^1,2^ This phenomenon results from the default protected wound-healing response to prolonged exposure to various liver injuries by different etiologies, including chronic viral hepatitis, nonalcoholic steatohepatitis, autoimmune diseases, and alcoholic liver disease.^3,4^ Activated hepatic stellate cells (aHSC) are the primary driver of liver fibrosis, producing approximately 90% of collagens during fibrosis development.^5,6^ Considering the prominent role of aHSCs in the pathogenesis of liver fibrosis, blocking pathways that promotes HSC activation is a promising therapeutic approach to treating the disease. Nuclear factor-kappa B (NF-κB) is the primary regulator of immune and inflammatory responses to infection and guards the aHSC against tumour necrosis factor-alpha (TNF-α) induced apoptosis. NF-κB is expressed in both qHSCs and aHSCs and after activation of HSC, the amount of NF-κB dramatically increases.^7^ This increase results in the transcription of interleukin-6 (IL-6), interleukin-8 (IL-8), intercellular adhesion molecule-1 (ICAM-1), and cyclooxygenase-2 (COX-2), and subsequently, the aHSCs become resistant to pro-apoptotic signals.^8^ NF-κB contributes to HSCs activation by mediating the synthesis of profibrogenic transforming growth factor beta-1 (TGF-β1). Lipopolysaccharides (LPS)-induced toll-like receptor-4 (TLR-4)/NF-κB signaling pathway consequenced in the reduction of bone morphogenetic protein and activin membrane-bound inhibitor (BAMBI, a TGF-β pseudo-receptor), which leads to enhancing TGF-β1-dependent activation of HSCs.^9^ In a feedforward loop manner, activation of TGF-β1/Smad signaling pathways interacts with the NF-κB signaling pathway.^10^

 In normal cellular conditions, NF-κB is complexed with the inhibitor protein IκB in the cytoplasm. During HSC activation, IκB becomes phosphorylated, which leads to its dissociation from NF-κB and subsequent degradation by the proteasome ^7^. Therefore, proteasome inhibition maintains NF-κB in complex with IκB despite its phosphorylation and subsequently prevents NF-κB activation and ameliorates liver fibrosis. Bortezomib (BTZ), a well-known proteasomal inhibitor widely applied for treating multiple myeloma and mantle cell lymphoma,^11,12^ demonstrated promising results in attenuating liver and renal fibrosis via suppressing NF-κB signaling and further TGF-β1 activation Conversely, inhibiting NF-κB signaling may exacerbate liver injury by promoting hepatocyte damage, which is a limitation of BTZ’s therapeutic approach against hepatic fibrosis.^13,14^ Moreover, the low solubility in water, poor specificity, and considerable toxicity are well-known drawbacks of BTZ.^15,16^ Therefore, explicitly and effectively targeting aHSCs might be a solution to overcome BTZ’s limitations in treating hepatic fibrosis.

 Due to their advantages in targeted drug delivery, controlled/sustained release, biocompatibility, and suitability for numerous water-insoluble drugs, synthetic polymeric nanoparticles (NPs) have increasingly been used to treat various diseases.^17,18^ The successful delivery of the therapeutic materials is associated with the applied NP system’s composition, structure, shape, size, and diverse and unique properties.^19^ Polylactic-co-glycolic acid (PLGA), a well-known biodegradable polymer, is approved by the Food and Drug Administration (FDA) for biomedical application in human and drug administration,^20^ which demonstrated non-toxicity through oral administration.^21,22^ Pegylated poly lactide-co-glycolide (PEG-PLGA) was co-formulated in the polymeric NPs to diminish the size and elevate the stability of NPs in blood circulation.^23^ Importantly, HSCs absorb approximately 90% of the vitamin A in the liver.^24^ To achieve improved accumulation in the liver, vitamin A-functionalized PEG-PLGA NPs might be an ideal BTZ drug delivery system, enhancing bioavailability and diminishing toxicity. The current study aimed to demonstrate the profound anti-fibrotic activity of BTZ-loaded PEG-PLGA–vitamin Ananoparticles in the carbon tetrachloride (CCl_4_) mouse model of liver fibrosis.

## Methods

###  Formulation and preparation of PEG-PLGA-VA + BTZ 

 PEG-PLGA-VA + BTZ were formulated and prepared using the emulsion solvent evaporation method. In brief, the PEG and PLGA polymers and BTZ (Organic phase) were rapidly added to polyvinyl alcohol (PVA) as an emulsifying agent (aqueous phase), which resulted in the immediate formation of an oil/water emulsion. Referring to the principle of the ‘similar phase dissolving method,’ the organic solvents evaporate under diminished pressure, transferring BTZ to the polymer-based hydrophobic core via hydrophobic interactions. This further solidifies the particles, shaping compacted NPs. Moreover, the presence of the emulsifier at the interface prevents the aggregation of NPs and separates the oil and water phases. This process leads to shaping NPs loaded with BTZ and VA based on hydrophobic interactions.

 PEG-PLGA-VA + BTZ were prepared in a nanoprecipitation manner. Briefly, the PEG-PLGA mixture in the ratio of 1:2 w/w was dissolved in 3 mL of ethanol/acetone mixture in the proportion of 1:2 v/v. Next, the solvent was added to 6 mL, 3% w/v of the PVA (aqueous solution), which contains both vitamin A (5 mg) and BTZ (5 mg). The resultant was stirred by an Ultrasonic probe (Qsonica, USA) (at 40% power, for 5 minutes), and the solvent was evaporated by vacuum at 40 ˚C. Following the evaporation, the centrifugation at 17 000 rpm for 30 minutes at room temperature results in the collection of the synthesized NPs. Next, NPs were washed with distilled water twice, resuspended again in distilled water, and frozen at -20 ˚C overnight. Finally, the NPs solution was lyophilized by applying freeze-dryer equipment (Millrock, USA).

###  Characterization of synthetic nanoparticles loading with drug

 Various methods were applied to investigate the characteristic of NPs. The geometry of the synthesized NPs was detected using scanning electron microscopy (SEM) (Zeiss LEO 1430VP, Zeiss, Germany). Dynamic light scattering (DLS) was utilized by applying a Zetasizer Nano ZS (Malvern, UK) to assess particle size distribution. The Fourier-transform infrared (FT-IR) spectra were used on a Bruker Tensor 270 spectrometer (Bruker, Germany). X-ray diffraction (XRD) assessment was achieved by a Bruker D8 Advance diffractometer (Bruker, Germany) applying CuKα radiation (λ = 1.542 A˚).

####  BTZ content and encapsulation efficiency 

 The drug content (DC) and encapsulation efficiency (EE) of the BTZ in the PEG-PLGA NPs were calculated by the HPLC method using an Xtimate® C18 column (25 cm × 4.6 mm, particle size: 5 µm, Welch Technology, Shanghai, China), acetonitrile (mobile phase A), and 0.1% phosphoric acid solution (mobile phase B).

 The injection volume was 20 µL, the temperature of the column was 37 °C, the flow rate was 0.5 mL/min, and the detection ultraviolet (UV) wavelength was 270 nm and maximum 30 Mpa pressure. For HPLC analysis, lyophilized NPs were accurately weighed and dissolved in 80% acetonitrile. The retention time of BTZ was approximately 17 min. Calibration curves were diagramed in a 1–100 µg/mL concentration range. The following formula calculated the DC and EE of the BTZ:


$$EE\%=\frac{Weight\;of\;encapsulated\;drug}{Weight\;of\;drug\;used}\times 100$$

$$DC\%=\frac{Weight\;of\;encapsulated\;drug}{Weight\;of\;nanoparticles}\times 100$$

###  In vitro cytotoxicity assessment 

 Human hepatic stellate cell line (LX-2) were seeded at a density of 1 × 10^4^ cells/well in a 96-well in 200 μL of complete Dulbecco’s modified eagle medium (DMEM) included 10% fetal bovine serum (FBS) with 1% penicillin/streptomycin at 37 °C in a humidified atmosphere containing 5 % carbon dioxide (CO2) for 24 hours.

 After that, the cells were treated with different concentrations (0, 5, 10, 20, 40, 80, and 100 µg/mL) of targeted NPs loaded with BTZ (PEG-PLGA-VA + BTZ) and without BTZ (PEG-PLGA-VA) in two different groups. After 48h treatment, a 3-(4,5-dimethylthiazol-2-yl)-2,5-diphenyl-2H-tetrazolium bromide solution (MTT) was added to each well with a final concentration of 0.5 mg/mL in phosphate-buffered saline (PBS) and incubated for 4 hours at 37 °C. After removing the medium, 100 µl of DMSO was added to each well to solubilize MTT-formazan. The absorbance was detected at 570 nm and 630 nm using a microplate reader (NanoDrop, Thermo Fisher, UK). MTT test was done in triplicate, and cell viability was normalized to that of cells without any treatment (the indicator of 100% cell viability).

####  In vitro assessment of NPs+VA uptake by fluorescent imaging

 The hepatoma cell line, HepG2 and LX-2 cells were seeded at a density of 5 × 10^4^ cells/well in 24-well tissue culture plates in a complete DMEM culture medium and at 37 °C with 5% CO2. After 48 hours, cells were treated with nontargeted NPs containing Rhodamine Red^TM^-X, and NPs targeted via VA loading Rhodamine Red^TM^-X for 24 hours, The final concentration was determined based on the results of the MTT assay, and the effective concentrations were selected as follows: two concentrations (20 mg and 80 mg) for HepG2 cells, and one concentration (20 mg) for LX-2 cells. At first, the medium was removed, and cells were washed with PBS twice. Next, cells were fixed with 5% formaldehyde for 20 minutes and then treated with Triton X-100 (0.3%,). 4’,6-Diamidino-2-phenylindole (DAPI), in the concentration of 20 µg/mL, was applied to stain the nuclei of the cells. Finally, the images were taken with a fluorescent microscope (Olympus, Japan).

###  In vivo study

 Thirty male BALB/c mice, 6-8 weeks old, weighing 20 ± 2, were purchased and acclimatized for 1 week before the beginning of the experiment procedure. The mice were maintained under standard laboratory conditions in a temperature and humidity-controlled environment with a 12 h light/dark period and fed adequate pellet food and water. Animal care and use were performed according to guidelines of Animal Research Ethics Committee of Tabriz University of Medical Science (IR.TBZMED.VCR.REC.1399.472).

####  Liver fibrosis mouse model establishment and treatment design

 To establish liver fibrosis, the mice were treated with 100 μL of 0.7% body weight in CCl4 dissolved in olive oil twice per week for 6 weeks. Next, the mice with fibrotic liver were randomly distributed into the following five groups, each containing five mice receiving treatment twice weekly for two weeks; A) mice treated with intravenous (IV) injection of targeted nanoparticle containing BTZ (BTZ + NP + VA), B) mice systematically received nanoparticle free BTZ, C) mice treated with non-targeted nanoparticle containing BTZ (BTZ + NP), D) mice treated with targeted nanoparticle without BTZ (NP + VA) and finally E) mice without any treatment and administered CCL4 during the treatment period as the positive control group. The duration of treatment for all groups takes 2 weeks. We also designed a negative control group as the number of the other groups by administrating PBS weekly during treatment time. 3 days after the last injections, all the mice were anesthetized, and blood and liver tissues were collected for the following investigations.

####  Histopathological assessment and immunohistochemical investigation

 The liver tissue was cut into small pieces and kept in a 10% formalin-saline solution to dehydrate overnight before being embedded in paraffin wax. 4µM thick liver sections were then stained with hematoxylin and eosin (H&E) to evaluate liver histopathological alterations using the METAVIR’s pathological scoring system.^25^ Moreover, Masson’s trichrome and Sirius-Red staining was applied to investigate the collagen fibers repositioning. The histopathological changes were observed using a light microscopy device (Inverted Microscope) (Olympus, Japan) Sirius-Red and α-SMA stained sections were semi-quantified for the percentage of the positive area utilizing ImageJ software 9 version 1.53n.

####  Bio distribution of NPs with vitamin A/rhodamine

 To investigate biodistribution, liver targeting, and estimate the maximum accumulation time, a volume of 1 mL of NPs soluble in PBS (Targeted with VA) and NPs (Nontargeted) was injected intravenously into animals and was traced after three-time points (0, 2, 4, and 6 hours). Rhodamine Red^TM^-X was applied as the fluorescence probe to visualize synthetized NPs. Kodak Image Station In-Vivo F/FX (Kodak, USA) was used for this assessment at λ ex = 596 nm and λ em = 755 nm. After 6h, mice were euthanized with 5% chloral hydrate; the liver, kidney, spleen, and heart were excised, and the presence of rhodamine Red^TM^-X in the desired organs was investigated by confocal microscope (Kodak, USA). All the pictures were investigated by the ImageJ/Fiji (version 1.50e)

####  Biochemistry investigations

 Serum biochemical assessment was done to detect liver dysfunction serum indicators. The plasma was collected by centrifugation at 4000 rpm/min for 15 min at 25°C. Next, the serum biochemical parameters level, including alanine aminotransaminase (ALT), aspartate aminotransferase (AST), and alkaline phosphatase (ALP), were measured by applying commercial kits (Diagnostic system, Germany) using the spectrophotometry device (Eon, Biotech, USA) at the 370 nm wavelength.

####  The expression of liver fibrotic-associated genes and BTZ target genes by qRT-PCR

 Initially, total mRNA was extracted from the liver tissues of different experimental groups using the Total RNA Purification Mini Kit according to the manufacturer’s instructions. Next, total RNA concentration was measured with the spectrophotometer and converted into cDNA by applying RevertAid First Strand cDNA Synthesis Kit the quantitative Reverse Transcription Polymerase Chain Reaction (qRT-PCR) was performed with SYBR Green I PCR Master Mix (Applied Biosystems) using the Rotor-Gene device (Qiagen, Germany) to evaluate fibrotic markers expression (*Collagen I*, *Collagen III*, and* α-SMA*) and BTZs targeted genes (*NF- κB*and *TGF-β*). Ribosomal protein L 13 (*RPL13)* is assumed as an internal control. Specific primer sequences designed by using Oligo7 primer analysis software (version 7.5) (Table 1). REST 2009 software (version 2.0.13) was used to assess the relative gene expression.

**Table 1:** Sequences of desired primers for assessment of target genes

**Target gene**	**Oligonucleotide sequence (5'- 3')**	**Gene ID**
*α -SMA *	F: GTCCCAGACATCAGGGAGTAA	11475
	R:TCGGATACTTCAGCGTCAGGA	
*Collagen I *	F: ACGCCATCAAGGTCTACTG	12824
	R:ACTCGAACGGGAATCCATC	
*Collagen III *	F: TGACTGTCCCACGTAAGCAC	12825
	R:GAGGGCCATAGCTGAACTGA	
*NF- κ B*	F: ATGGCAGACGATGATCCCTAC	18033
	R:TGTTGACAGTGGTATTTCTGGTG	
*TGF- β*	F: TGGAGCAACATGTGGAACTC	21803
	R:TGCCGTACAACTCCAGTGAC	
*RPL13*	F: AGCAGATCTTGAGGTTACGGA	270106
	R:GGCATGAGGCAAACAGTCT	

###  Western blotting

 The protein content of liver specimens was extracted by applying radioimmunoprecipitation assay (RIPA) lysis buffer according to the manufacturer’s instructions. The total protein concentrations in sample lysate were measured using the bicinchoninic acid (BCA) protein assay (DNAbiotech, Iran). Proteins (40 μg/lane) were electrophoretically separated on the 10% sodium dodecyl sulfate-polyacrylamide gel electrophoresis (SDS-PAGE) and transferred to polyvinylidene difluoride (PVDF) membranes.

 The membrane was then blocked with Tris-buffered saline (TBS) containing 5% skim milk and incubated at 4°C for 16 hours. After that, the membrane was probed with the primary antibodies of TGF-β (1:1000 dilution), NF-κB (1:2000 dilution), Smad3 (1:1500 dilution), phosphorylated Smad3 (p-Smad3) (1:1000 dilution), and beta-actin (β-actin) (Invitrogen, USA) as an internal control. This step was followed by incubation at room temperature for 1 h and washing with TBS containing Tween 20 (TBS-T) buffer. Finally, protein bands were visualized using the enhanced chemiluminescence (ECL) system (Merck, Germany). ImageJ (version 1.50e) software was applied to quantitatively analyze western blots.

###  Statistical analysis

 Statistical analysis of results was performed with Prism software version 9 (GraphPad Software, USA) and SPSS (version 24). One-way analysis of variance (ANOVA) test and Bonferroni’s multiple comparisons were used to compare the data from the different experimental groups for the respective studies. Data were indicated as mean ± standard error of the mean (SEM), and the *P *value ≤ 0.05 was considered significant.

## Results

###  Characterization of synthesized targeted NPs containing VA and BTZ

**Figure 1:**
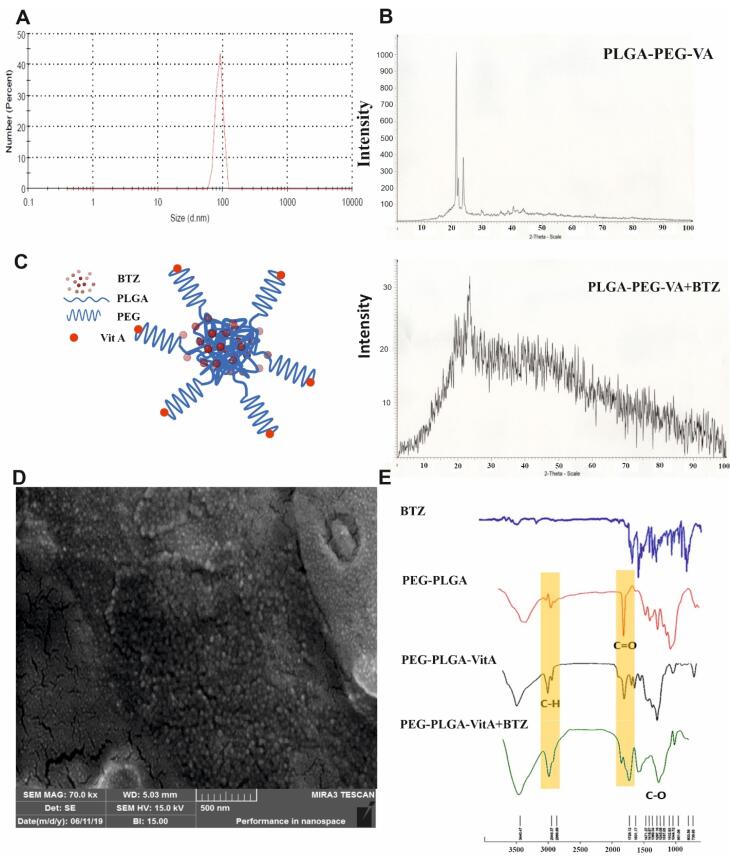
**Characterization of synthesized PEG-PLGA-VA+BTZ NPs. A)** The particle size of BTZ + NP + VA was measured by the DLS method. **B) **XRD pattern of PEG-PLGA-VA NPs and PEG-PLGA-VA NPs loaded with BTZ.** C) **Aschematic and conceptual figure of BTZ entrapment is shown in the structure of NPs. **D) **SEM images of the NPs (the scale bar represents 100 nm).** E)** FT-IR of spectra of BTZ, PEG-PLGA, PEG-PLGA-VA, and PEG-PLGA-VA + BTZ NPs. PLGA: Poly lactic-co-glycolic acid, VA: Vitamin A, BTZ: Bortezomib. NP; Nanoparticle, VA; Vitamin A

 BTZ + NP + VA nanoparticles were synthesized using the emulsion solvent evaporation method. Dynamic light scattering (DLS) using a Malvern Instrument (Malvern, UK) was applied to assess the size of the synthesized NPs. Considering the results, the average size of BTZ + NP + VA was about 93 ± 0.69 nm. Particle size is a critical factor that impacts the hepatic uptake of NPs. In the current study, the BTZ + NP + VA size was below the determined limit of < 200 nm for passive targeting of the liver (Figure 1).

 Next, the structural properties of synthesized NPs were investigated using XRD analysis; the results are shown in Figure 1B. The XRD pattern of NPs containing PEG-PLGA-VA revealed that crystal plates in the sample could be detected at the peak of 2θ at 21°, 22°, and 24°. Crystal plates were detected in samples of PEG-PLGA-VA NPs loaded with BTZ at the peak of 2θ at 22°.

 FT-IR analyses were performed to identify possible interactions. FT-IR investigations demonstrated that BTZ in the formulation had kept its parent chemical structure. As it is evident in Figure 1E, the bands around 1720 cm^-1^ and 1631 cm^-1^ correspond to C = O stretching frequencies. The absorption of aromatic C-H stretches was observed around 2949 cm^-1^. Moreover, strong absorptions were noted at 1297 cm⁻¹ which is related to the C–N group. The results confirmed that although a small shift in frequencies is observed, all the characteristic peaks of BTZ are present in PEG-PLGA-BTZ + NP + VA, indicating that BTZ was physically entrapped in the synthetized NPs (Figure 1E).

 SEM was applied to investigate the morphology of the synthesized NPs containing BTZ. The data indicated a spherical shape for synthesized PEG-PLGA-VA + BTZ NPs with uniform and relatively narrow particle distribution (Figure 1D). Additionally, a mean diameter of 50.03 nm was obtained for the synthetized NPs by statistically assessing the SEM image.

###  Determination of BTZ in prepared NPs using HPLC

**Figure 2:**
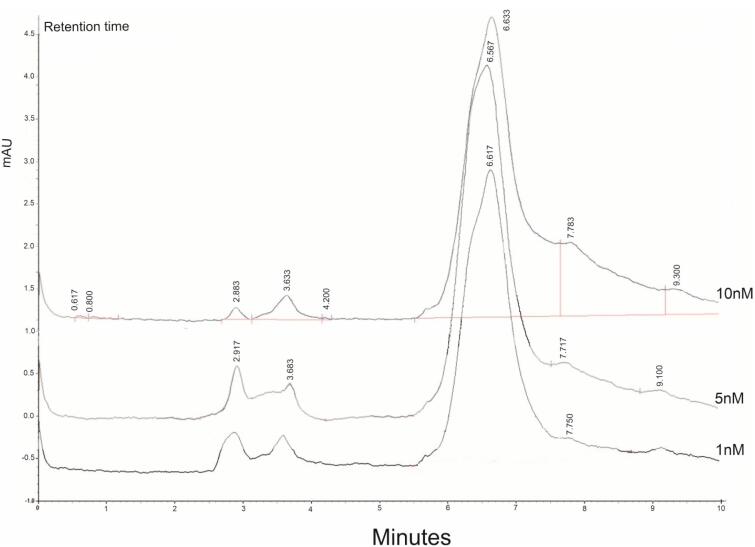
**The representative Chromatogram of BTZ. **To evaluate the content of drug encapsulated by NPs HPLC method was used. The absorption of different concentrations of BTZ was shown then the calibration carve with the accumulated concentration of the drug was done (1nM, 5nM, 10nM)

 The content of BTZ encapsulated in synthesized NPs was investigated using the HPLC method to evaluate the efficiency of BTZ loading in NPs. First, five standard samples of BTZ with concentrations of 1, 5, 10, 50, and 100 µg/mL were prepared and injected into the device (Figure 2). A calibration curve was calculated based on the chromatograms. Using the equation resulting from the standard curve and the drug absorbance, the DC in 1 mg of NPs was found to be 63.98 µg. Consequently, the DC was calculated to be 6.3%.

###  Impact of BTZ loaded in NP + VA on viability of LX-2 and HepG2 cells

**Figure 3:**
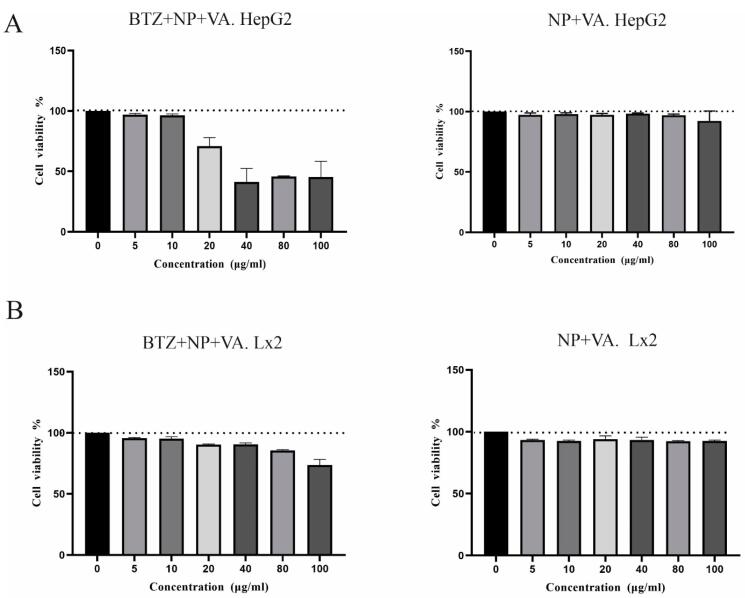
**Cytotoxicity of different doses of targeted NPs containing BTZ or not on LX-2 and HepG2 cell lines. A) **The viability of HepG2 cells was analyzed by MTT assays after treatment with different concentrations of BTZ entrapped in targeted NPs (0, 5, 10, 20, 40, 80, and 100 µg/mL) for 48 h and drug free NPs**. B**) The viability of LX-2 cells was analyzed by MTT assays after treatment with different concentrations of targeted NPs with VA containing BTZ A (0, 5, 10, 20, 40, 80, and 100 µg/mL) for 48 h and drug free NPs. The data represented the mean ± SEM of two independent experiments.NP; Nanoparticle, VA: Vitamin A, BTZ: Bortezomib

 The MTT assay was used to assess the cytotoxicity of BTZ encapsulated in targeted NPs with VA as well as drug-free targeted NPs solution in various concentrations (0, 5, 10, 20, 40, 80, and 100 µg/mL) on the LX-2 and HepG2 cell lines. The MTT investigation was performed in triplicate for all the studied concentrations. The results for the HepG2 line revealed that with BTZ entrapped in NP + VA at concentrations of 5 to 20µM, more than 90% of the cells were viable, and this viability decreased to approximately less than 50% in concentrations of 40, 80, and 100 µg/mL (Figure 3A). Treating the cells with all the desired concentrations of synthesized NP + VA without BTZ (0, 5, 10, 20, 40, 80, and 100 µg/mL) did not exhibit any significant impact on the viability of the cells, which indicates that administration of NP + VA is not cytotoxic for HepG2 cells even in the highest applied concentration (100 µg/mL) (Figure 3A). Regarding the LX2 line, NP + VA with or without BTZ had no significant impact on cell viability from the lowest concentration (5µg/mL) to the highest one (100 µg/mL) (Figure 3B).

###  Cellular uptake of NPs targeted with vitamin A by HepG2 and LX-2 cell lines

**Figure 4:**
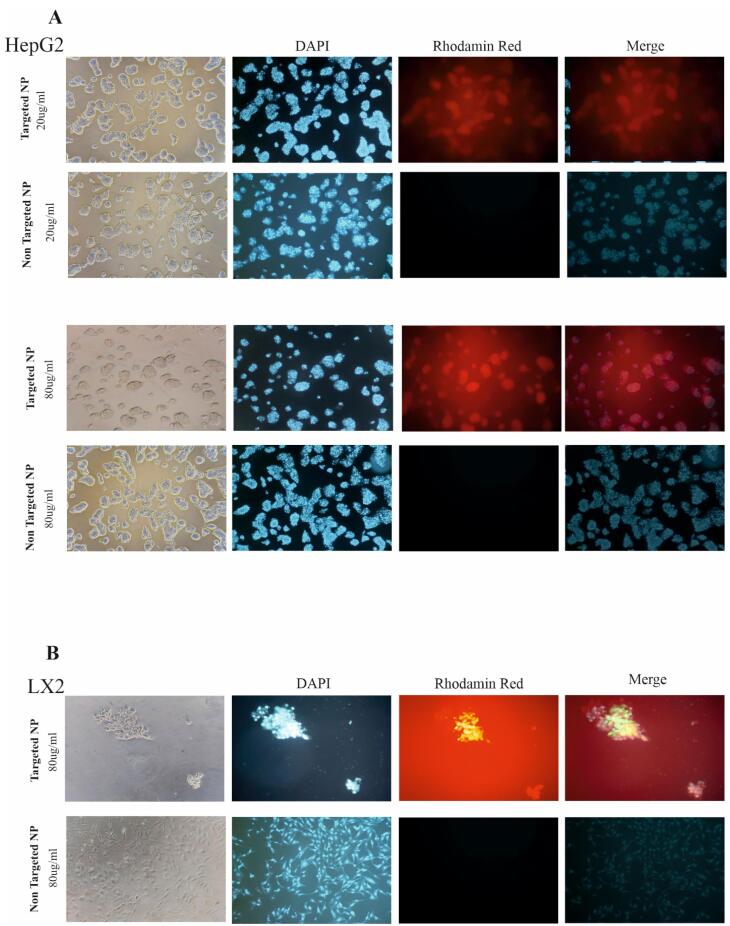
**Cellular uptake of targeted NPs and no targeted NP by confocal microscopy.** A) Represented images of targeted NPs and NPs without targeted administration with different concentrations of 20 mg and 80 mg in HePG2 cells. B) Represented images of targeted NP and targeted free NPs administration with the concentration of 20 mg in LX-2 cell line. DAPI staining for the nucleus is seen in blue. Images are represented at 100x magnification

 The cellular uptake capacity of the synthesized NPs alone and in combination with vitamin A was investigated by fluorescent microscopy. As shown in Figure 4, both HepG2 and LX-2 cells internalized substantial amounts of Rhodamine Red^TM^, the BTZ substitute, when administered with NP + VA at concentrations of 20 µg/mL and 80 µg/mL for HepG2 cells, and 80 µg/mL for LX-2 cells, compared to NP-treated cells. Confocal images demonstrated that modifying NPs with vitamin A enhanced the internalization of the synthesized NPs into the cytoplasm of HepG2 (Figure 4A) and LX-2 cells and helped raise the consequences of targeted therapy BTZ (Figure 4B).

###  Biodistribution of BTZ + NP + VA in mice

**Figure 5:**
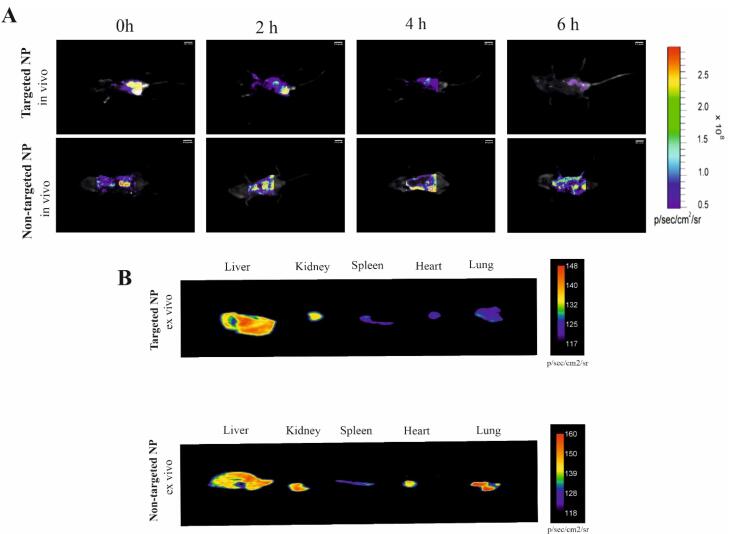
**Bio distribution comparison of targeted NP and NPs. **Mice were intraperitoneally injected with desired NPs-labeled Rhodamine Red^TM^-X probes and then imaged**. A) **Time-lapse fluorescence imaging (0 to 6 h) and ex-vivo imaging of the organs after 6h administration of targeted NPs and targeted free NPs.** B) **Time-lapse fluorescence imaging (0 to 6 h) and ex-vivo imaging of the organs after 6h administration of nontargeted NPs as well as targeted NPs. The Vitamin A conjugation into NPs exhibited a good performance both in vivo, thus paving the way for that NP modified Nano system for targeted liver therapy. NP; Nanoparticle

 To elucidate the biodistribution of synthesized NPs, mice were intraperitoneally injected with VA NPs (targeted) and NPs (nontargeted, conjugated with Rhodamine Red^TM^-X). As clearly illustrated in Figure 5, real-time fluorescence imaging at the initial distribution period (Timepoint 1 = 0) revealed that fluorescence signals were exhibited from the abdominal area in mice administered both targeted and nontargeted NPs. From the second time point (2 h) to the last examined time point (6 h), fluorescence signals were detected from mice treated with targeted NPs showing a gradual tendency towards the liver zone, whereas the signal distribution observed in mice treated with nontargeted NPs was detected from a different site in the abdominal area. The most significant difference between the targeted and nontargeted treated groups was observed 6h after injection (Figure 5A). Further ex vivo investigation demonstrated enhanced accumulation of NP + VA in the liver compared to other investigated organs (heart, kidney, pancreas, and spleen), At the same time, a constant level of NPs was detected in different organs of the untreated mice (Figure 5B).

 Considering the fluorescence imaging results, greater accumulation of nontargeted NPs in the liver zone could be achieved by decorating the drug delivery system with a coating of vitamin A.

###  BTZ + NP + VA attenuated liver fibrosis from microscopic and histological aspects

 To further validate the potential of BTZ + NP + VA in alleviating liver fibrosis, we conducted a liver biopsy and histological evaluation, which are widely recognized as the gold standard methods for assessing liver fibrosis.

**Figure 6:**
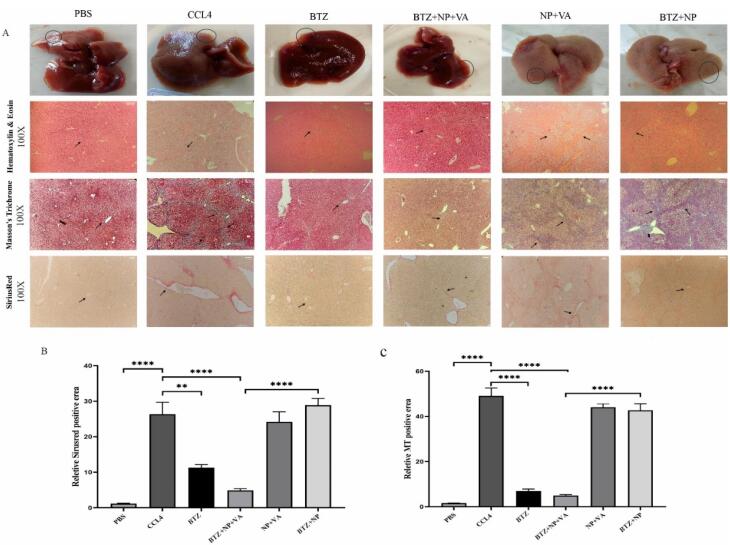
**Microscopic, histopathological assessment, and liver fibrosis grading. A)** Macroscopic observation of liver specimens and Histopathological assessment of hepatic tissues by H&E staining (magnification 100x), Masson’s Trichrome staining (magnification 100x), and Sirius-red staining (magnification 100x). The arrows indicate the thickness of the fibrous septa. **B) **Semi-quantitative analysis of Sirius-red staining to measure collagen deposition, and **C)** Semi-quantitative analysis of Masson’s Trichrome staining to investigate the grade of hepatic fibrosis. Data are shown as mean + SEM (n = 5). ANOVA test and Bonferroni’s multiple comparisons were used for statistical analysis. ** value < 0.01 and **** P value < 0.0001. NP; Nanoparticle, VA; Vitamin A; CCL4: Carbon Tetrachloride, and PBS; Phosphate buffer saline, BTZ: Bortezomib

 The liver tissue from the positive control group (CCL4 recipients) showed a fractured surface with dark red color. In contrast, the liver tissue from the healthy control group (PBS recipients) had a smooth texture and pale red color, as observed by microscopic examination. The microscopic observations of drug-free NPs targeted with VA and BTZ + NPs without VA groups detected no considerable recovery from liver damage. Conversely, both the NPs consisting of BTZ + VA and systematic BTZ administration reduced liver damage and improved liver structure (Figure 6A).

 Hematoxylin and eosin (H&E) and Masson’s trichrome staining demonstrated the infiltration of inflammatory cells around the portal area, hepatocyte degeneration, disorganized hepatic cords and central veins, and collagen deposition in the NP + VA and BTZ + NP groups similar to the CCl₄-recipient group. Moreover, administration of NPs containing both VA and BTZ and systemic BTZ showed a near-typical hepatic structure with few collagen fibers in the portal area. The semi-quantitative evaluation of Masson’s Trichrome staining indicated that inflammatory grade and fibrosis stage significantly declined in both BTZ + NP + VA and systematic BTZ-recipient groups compared with the CCL4-recipient group. Moreover, the inflammatory grade and fibrosis stage reduction were higher in the BTZ + NP + VA recipient group (Figure 6B).

 Consistent with the Masson’s trichrome staining results, the Sirius-red staining assessment of the NP + VA and BTZ + NP groups revealed the accumulation of collagen fibers around the portal area, just as the CCL4-recipient group, whereas a notable decline in collagen fibers was observed in the BTZ + NP + VA and systematic BTZ recipient groups. The Sirius-red semi-quantitative data confirmed that the administration of both BTZ + NP + VA and systematic BTZ caused a significant decrease in collagen fibers accumulation compared to the CCL4-recipient groups with a *P *value < 0.0001 and *P *value < 0.01, respectively. As indicated from the results, BTZ + NP + VA more effectively reduced collagen accumulation (Figure 6C).

**Figure 7:**
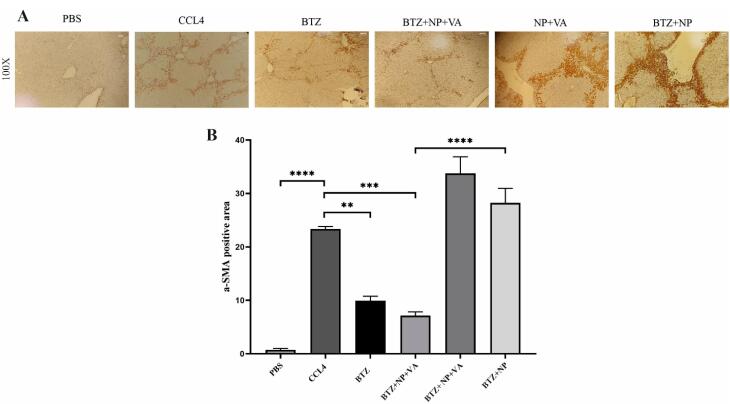
**Immunohistochemistry staining against **α**-SMA in. A) **the microscopic observation of liver specimens immunohistochemy staining againstα-SMA (magnification 100x)**. B) **Semi-quantitative analysis of α-SMA expression. Data are shown as mean + SEM (n = 5). ANOVA test and Bonferroni’s multiple comparisons were used for statistical analysis. **P-value < 0.01 and ****P-value < 0.0001. NP; Nanoparticle, VA; Vitamin A; CCL4: Carbon Tetrachloride, and PBS; Phosphate buffer saline, BTZ: Bortezomib

 Immunohistochemical staining for α-SMA expression in liver tissue revealed a significant increase in α-SMA expression in the CCL4-treated livers compared to controls (Figure 7). The same result occurred for the drug-free NPs (NP + VA) and the group treated with non-targeted NPs (BTZ + NP). The results demonstrate that drug delivery plays a fundamental role in the efficacy of BTZ + NP + VA. In line with these results, systemic BTZ could suppress the expression of α-SMA. The semi-quantitative results confirmed the effect of BTZ + NP + VA on liver fibrosis with a *p*-value < 0.0001.

 Overall, BTZ + VA carried by NPs indicated considerable potential to prevent inflammation and collagen deposition, restrict fibrosis progression, and further attenuate liver fibrosis in mice with CCl₄-induced liver fibrosis.

###  Systematic BTZ and BTZ loaded in NP + VA influence liver functions

**Figure 8:**
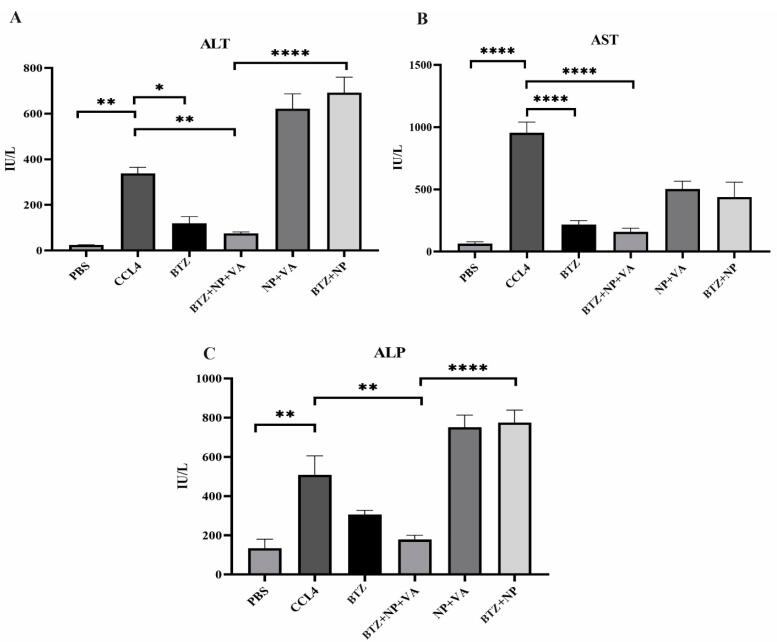
**Impact of different treatments in liver biochemical parameters.** A) The level of circulating ALT (IU/L), B) The level of circulating AST (IU/L), and C) The level of circulating ALP (IU/L). Data were represented as mean ± SEM (n = 5). ANOVA test and Bonferroni’s multiple comparisons were performed for statistical analysis. * P value < 0.05, **P value < 0.01, and **** P value < 0.0001. NP; Nanoparticle, VA; Vitamin A; CCL4: Carbon Tetrachloride, and PBS; Phosphate buffer saline, BTZ: Bortezomib

 The circulating levels of ALT, AST, and ALP were measured to investigate the extent of liver injury. The activity of both ALT and AST serum levels was considerably increased in the CCL4-recipient group in comparison with the PBS-recipient group with a *P *value < 0.01 and a *P *value < 0.0001, respectively (Figure 8). Administration of both BTZ + NP + VA and systematic BTZ revealed an apparent decrease in serum levels of ALT compared with the CCL4-recipient group with *P* value < 0.01 and *P *value < 0.05, respectively, returning to the normal level (Figure 8A). Investigation of AST revealed that the serum activity of the desired enzyme was reduced in the BTZ + NP + VA and systematic BTZ-recipient groups compared with the CCL4-recipient group by *P* value < 0.0001. Also, treatment with drug-free NP + VA and BTZ + NP did not significantly impact this parameter of liver function (Figure 8B).

 Additionally, as with the ALT and AST investigations, the administration of CCL4 resulted in a sharp rise in the ALP serum level compared with the PBS-recipient group (*P* value < 0.01). Treatment with BTZ + NP + VA caused a considerable decrease in ALP circulating levels compared with the CCL-4 recipient group with *P* value < 0.01. The other treatment groups did not show significant effects on ALP serum levels (Figure 8C).

 These results indicated that targeted NPs loaded with BTZ could alleviate CCl4-induced liver injury, promoting a return to the expected normal level of serum liver damage parameters.

###  BTZ + NP + VA suppresses the crosstalk between TGF-β1/Smad3 and NF- κB signaling pathways 

**Figure 9:**
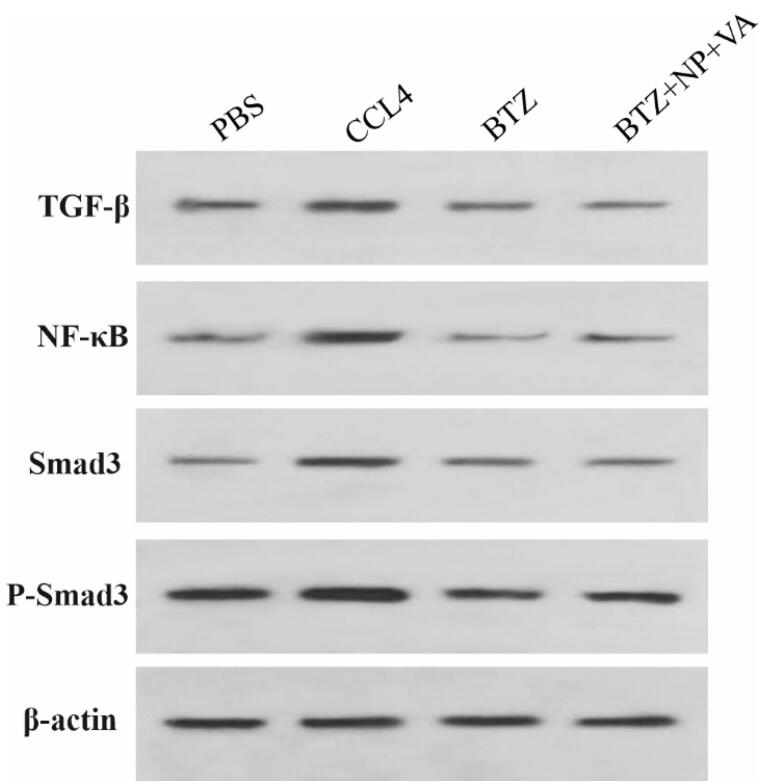
**BTZ+NP+VA inhibit HSC activation in liver fibrosis induced by CCL4**. Western blot analysis was performed for TGF-β, NF-κB, Smad 3, and p-Smad 3 proteins. β-actin was exploited as the internal control. TGF-β; Transforming growth factor-beta, NF-κB; Nuclear factor-kappa B, p-Smad 3; phosphorylated Smad 3, β-actin; Beta-Actin, NP; Nanoparticle, VA; Vitamin A; CCL4: Carbon Tetrachloride, and PBS; Phosphate buffer saline, BTZ: Bortezomib

 The inhibitory impacts of BTZ + NP + VA on the TGF-β1/Smad3 and NF-κB signaling pathways were confirmed *in vivo*. Western blot investigation revealed that intoxication with CCL4 caused a remarkable elevation in TGF-β, NF-κB, Smad 3, and p-Smad 3 protein levels compared with the PBS-recipient group. At the same time, administration of both NPs containing VA + BTZ and systematic BTZ noticeably diminished the protein levels of TGF-β, NF-κB, Smad3, and p-Smad3 compared to the CCL-4 recipient group. This reduction caused the desired protein levels to return to approximately normal levels (Figure 9). Altogether, the combined data approved the potential of VA + BTZ synthesized NPs in surpassing the interaction between TGF-β1 and NF-κB signaling pathways and further attenuating hepatic fibrosis in a CCL4-induced mouse model of hepatic fibrosis.

###  Declines in liver genes expression in vitro and in vivo assessment after treatment with BTZ + NP + VA and BTZ

**Figure 10:**
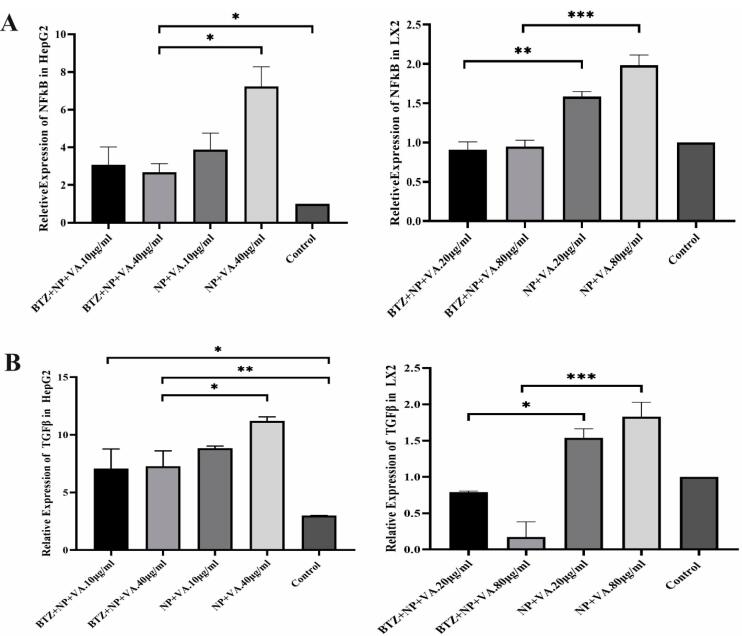
**Relative expression of TGF-β and NF-κb in two hepatic cell lines**. **A**) Differential Expression of NF-κB in HepG2 and LX2 lines in two concentrate of targeted NP loaded with BTZ and BTZ free targeted NPs. **B**) different expression of TGF-β in HepG2 cell line and LX2 line during treatment with targeted NP with BTZ and without BTZ. Data were represented as mean ± SEM (n = 5). ANOVA test and Bonferroni’s multiple comparisons were performed for statistical analysis. * P value < 0.05, ** P value < 0.01, and **** P value < 0.0001.TGF-β; Transforming growth factor-beta, NF-κB; Nuclear factor-kappa B. NP; Nanoparticle, VA; Vitamin A; CCL4: Carbon Tetrachloride, and PBS; Phosphate buffer saline, BTZ: Bortezomib

 The effect of NPs containing BTZ on TGF-β and NF-κB expression in HepG2 and LX2 cell lines was evaluated through qRT-PCR. The selected concentration was based on our previous MTT assay representing low and high doses for each cell line (Figure 10).

**Figure 11:**
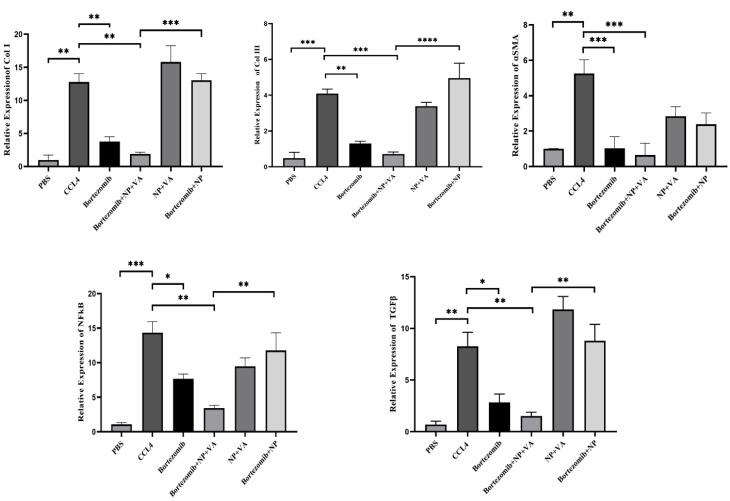
**Differential expression of selected fibrotic markers and BTZ targeted genes. A)** Quantifying Collagen I, Collagen III expression, and α-SMA gene expression by qRT-PCR. **B) **Alteration in gene expression of NF-κB and TGF-β. The expression of desired genes was normalized to the housekeeping gene, RPL13. Bars represented the mean ± SEM of three independent experiments. ANOVA test and Bonferroni’s multiple comparisons were performed for statistical analysis. * P value < 0.05, ** P value < 0.01, *** P value < 0.001, and **** P value < 0.0001. NF-κB; Nuclear factor-kappa B, TGF-β; Transforming growth factor-beta, α-SMA; Smooth muscle alpha-actin, NP; Nanoparticle, VA; Vitamin A; CCL4: Carbon Tetrachloride, and PBS; Phosphate buffer saline, BTZ: Bortezomib

 To address the role of BTZ on NF-κB and TGF-β signaling pathways in the HSCs activation process and further liver fibrosis, first, the expression levels of fibrosis-associated genes, including collagen I, collagen III, and α-SMA, were assessed by qRT-PCR. As indicated in Figure 10, a considerable elevation in the mRNA expression levels of collagen I by *P* value < 0.01, collagen III by *P* value < 0.001, and α-SMA by *P* value < 0.01 was observed in CCL4-recipient mice compared to the PBS-recipient group. No statistically significant alteration in the desired gene expression was detected after treatment with drugless VA + NPs and nontargeted NPs carrying BTZ. In contrast, administration of both BTZ + NP + VA and systematic BTZ significantly diminished the mRNA expression levels of collagen I (*P* value < 0.01), collagen III (*P* value < 0.001, *P* value < 0.01, respectively), and α-SMA (*P* value < 0.001) compared with the CCL4-recipient group (Figure 11).

 Next, we assessed the NF-κB and TGF-β expression levels of the treatment groups. Like the fibrotic-related gen examination, CCL4 exposure increased the expression levels of both NF-κB and TGF-β by *P* value < 0.001 and *P* value < 0.01, respectively, compared with the PBS-recipient group. Administration of VA + NPs and BTZ + NP to the CCL4-liver fibrosis-induced mice showed no great potential for altering either NF-κB or TGF-β expression level. Conversely, treatment with BTZ + NP + VA and systematic BTZ had tremendous effects in reducing NF-κB (*P* value < 0.01, *P* value < 0.05, respectively) and TGF-β (*P* value < 0.01, *P* value < 0.05, respectively) expression levels (Figure 11).

 Combined results in the group treated with BTZ loaded in targeted NPs with VA, indicated a tremendous anti-fibrotic capacity by suppressing HSCs activation and reducing the expression of NF-κB and TGF-β, further ameliorating liver fibrosis in the CCL4-induced mouse model.

## Discussion

 BTZ (Velcade®), a dipeptidyl boronic acid, plays a vital role in inhibiting the 26S proteasome, a key component of the ubiquitin-proteasome pathway responsible for degrading proteins, including cell-cycle regulators and transcription factors. By selectively and reversibly inhibiting the 26S proteasome, BTZ can manipulate various cellular pathways related to protein degradation and intracellular protein regulation. Clinical trials applying BTZ solo or combined with other anti-cancer agents in phases I, II, and III on non-Hodgkin lymphoma, myeloid leukemia, and multiple myeloma patients have demonstrated promising responses, considering response rate, time to progression, and survival.^26-28^ Several investigations have shown the potential of BTZ for treating fibrosis, such as kidney fibrosis.^29,30^ BTZ’s potential antifibrotic actions have attracted interest in fatty liver and fibrosis correlated with inflammation. Despite the significant BTZ therapeutic approaches, many of the patients who have received BTZ therapy experience adverse drug reactions, including fatigue, diarrhea, constipation, peripheral neuropathy, and hypotension. One of the reasons for these diverse side effects could be explained by the extensive BTZ biodistribution to almost all organs, which participate in the failure of the treatment or discontinuation. Hence, an appropriate drug delivery platform may result in increasing the accumulation of BTZ at the critical target site while reducing the occurrence of the putative side effects.^31-33^

 Although the mechanisms of BTZ function in liver fibrosis remain incompletely understood, it exerts its antifibrotic action mainly by inhibiting the NF-κB pathway components associated with TGF-β1 during the inducing apoptotic process. A previous study by Anan et al showed that proteasome inhibition by BTZ is a potential therapeutic approach to inducing HSCs apoptosis and further inhibiting hepatic fibrosis in the LX-2 cell line and bile duct-ligated (BDL) mice.^34^ The apoptosis process was shown to be induced by inhibition of NF-κB pathways, one of the main pathways involved in the progression of liver fibrosis.^35^ Moreover, the NF-κB pathway is related to TGF-β1 pathways in a feed-forward loop manner via diminishing the expression of BAMBI through the LPS-induced TLR-4/ NF-κB signaling pathway.^36^ Cumulative evidence considers TGF-β1 as one of the essential fibrotic cytokines. After the phosphorylation of type II and type I receptors, the intracellular substrates, the SMAD 2 and 3 proteins, will be phosphorylated. This complex translocates from the cytoplasm to the nucleus, and primes extend the matrix component’s expression, including collagen type I, collagen III, fibronectin, and proteoglycans. In the current investigation, we mainly assessed the impacts of both BTZ in VA-coupled NPs and systematic BTZ administration on CCL4-induced hepatic fibrosis mice. However, we hoped that targeted therapy with sustained release could live up to our expectations about the antifibrotic effects of the drug. PEG-PLGA nanoparticle was selected due to the high power of hydrophobic interaction with BTZ. Related to NPs, previous articles showed PEG-PLGA’s power to envelop drugs with high hydrophobic interactions.^37^ Sample preparation was evaluated using different ratios of drug and NPs with optimal VA. SEM, DLA, and FTIR tests confirm the shape, and drug-loaded NPs targeted the liver via VA.

 At first, we assessed ALT, AST, and ALP serum levels to evaluate liver function, in which the release of the mentioned enzyme rises into the bloodstream from the injured liver during fibrosis progression. Based on our results, the serum levels of ALT, AST, and ALP diminished after treatment with systematic BTZ and BTZ in VA-coupled NPs. The improvement of liver dysfunction was more significant in the BTZ and the VA-coupled NPs-recipient groups. The possible mechanism by which BTZ reduces the serum levels of liver dysfunction biomarkers is the diminishing hepatocyte nuclear factor−1α-induced promoter activation of cytochrome P450 2E1.^38^

 Moreover, Anan et al demonstrated in their study that BTZ treatment significantly reduced the serum level of ALT and further attenuated hepatic injury through BTZ-treated BDL C57/BL6 mice.^39^ Conversely, in the study of Saeki et al, BTZ administration did not show efficient potential to modulate AST and ALT in choline-deficient L-amino acid-defined (CDAA)-recipient Wistar rats.^40^ This discrepancy in study results may be attributed to the number and amount of BTZ injected in preclinical models. The histopathological examination data confirmed and supported our biochemical results. H&E and Masson’s trichrome staining indicated a considerably diminished liver bridging fibrosis and collagen deposition. Sirius-red observation revealed a substantial decrease of fibrous and collagen deposition patterns in BTZ in VA-coupled NP-recipient mice.

 Investigations demonstrated that apoptosis induction in transformed cells by proteasome inhibitors depends on the NF-κB activation.^41^ The classic complex of NF-κB is the p65-p50 that is inhibited with IkB, the best-known NF-κB inhibitor in normal and unstimulated conditions.^42^ IkBα is one of the target genes after activation of NF-κB in aHSCs, and two approaches are adopted by these cells to prevent the transcription of this NF-κB inhibitor. First, the activation of aHSCs coincides with the expression of CBF1, and this protein, in complex with MeCP2, targets the IkBα promoter and suppresses the expression of this gene. Therefore, the levels of NF-κB become abundant in aHSCs.^43,44^ In the second approach, in aHSCs, another form of IkB in a hyperphosphorylated form with a higher affinity to NF-κB is expressed with no inhibitory function. Hence, NF-κB would remain in an active state. Proteasome inhibition prevents IkB degradation, thereby keeping NF-κB complexed to this inhibitory protein despite its phosphorylation.^45,46^ In our study, the expression level of NF-κB was significantly reduced in mRNA and protein levels in both systematic application and BTZ in VA-coupled NPs treated mice.

 Similarly, Anan et al investigated the impacts of BTZ on the LX-2 cell line and revealed a significant reduction in the NF-κB expression after treatment with BTZ.^34^ The same results were observed in the Helerband study, which demonstrated NF-κB inhibition by BTZ in rat HSCs. Consistent with in vitro investigations, Anan and colleagues’ investigation of BTZ administration in BDL mice showed a decrease in NF-κB expression. This result suggests a reduction in activation of HSCs by BTZ.^34^ To further show the impacts of BTZ on TGF-β-dependent HSCs activation, the expression of TGF-β was assessed. Our in vitro and in vivo results revealed a considerable reduction in TGF-β expression. Also, based on the Western blot analysis, the protein levels of phosphorylated Smad3 and Smad3 were diminished after the administration of BTZ in VA-coupled PEG-PLGA. Consistently, several investigations have reported that BTZ prevents tissue fibrosis in the lungs and skin by suppressing TGF-β1 in preclinical models.^47,48^

 On the flip side, the impacts of BTZ on the other cell types in the liver must also be considered. For example, inducing apoptosis in hepatocytes is directly associated with increased inflammation and hepatic fibrosis.^49^ Also, most patients receiving BTZ therapy experience dose-dependent adverse side effects.^50^ Hence, we decreased the amount of BTZ on a scale of one-twentieth, which significantly showed promising results that could overcome all the previously mentioned barriers limiting medicine use. The present study’s in vitro and in vivo experiments consistently indicated that BTZ in VA-coupled NPs improved hepatic fibrosis. The activation marker of HSCs, α-SMA, was downregulated after BTZ intervention in mRNA and protein levels. The expressions of other fibrosis markers, procollagen types I and III, decreased, consistent with the amelioration of liver fibrosis considering the reduced accumulation of collagen content. It should be mentioned that the systematic application of BTZ also showed promise in reducing the clinical symptoms of hepatic fibrosis to a certain extent. Considering all the results, the loading of BTZ in VA-coupled PEG-PLGA NPs demonstrated promising outcomes in overcoming the clinical limitations associated with toxicity, low water solubility, unstable properties, and low concentration at the target site.

 In general, our results suggest that targeted delivery of proteasome inhibitors can effectively suppress liver fibrosis architecture and reduce the expression of genes related to ECM. Our study demonstrates that BTZ can significantly inhibit fibrotic liver through the TGF-β1 and Smad signaling pathways, even at lower concentrations when delivered through NPs. Targeted delivery of BTZ reduces its side effects, improves the fibrotic architecture of the liver, and reduces the severity of fibrosis.

## Conclusion

 In general, our results suggest that targeted delivery of proteasome inhibitors can effectively suppress liver fibrosis architecture and reduce expression of genes related to ECM. Our study demonstrates that BTZ can significantly inhibit fibrotic liver through the TGF-β1 and Smad signaling pathways, even at lower concentrations when delivered through NPs. Targeted delivery of BTZ not only reduces its side effects but also improves the fibrotic architecture of the liver and reduces the severity of fibrosis

## Competing Interests

 None declared.

## Ethical Approval

 Animal care and use were performed according to guidelines of Animal Research Ethics Committee of Tabriz University of Medical Science (IR.TBZMED.VCR.REC.1399.472).
